# Supplementary material for: Secondary grain boundary dislocations alter segregation energy spectra
Source: Nat Commun. 2025 Sep 25;16:8422. doi: 10.1038/s41467-025-64265-6 (PMC12462497; doi:10.1038/s41467-025-64265-6)
Supplement: Supplementary file 1 — Supplementary Information [file 41467_2025_64265_MOESM1_ESM.pdf]

Supplementary Information for Secondary Grain Boundary Dislocations Alter Segregation Energy Spectra

Xinren Chen[1], William Gonçalves[2], Yi Hu[1], Yipeng Gao[1], Patrick Harrison[3], Gerhard Dehm[1], Baptiste Gault[1,4], Wolfgang Ludwig[2], Edgar Rauch[3], Xuyang Zhou[1*] and Dierk Raabe[1]

[1]Max-Planck-Institut for Sustainable Materials, Max-Planck-Straße 1, Düsseldorf, 40237, Germany.
[2]Université Lyon I, MATEIS, INSA Lyon, CNRS UMR 5510, Villeurbanne, 69621, France.
[3]Université Grenoble Alpes, Grenoble INP, SIMaP, Grenoble, F-38000, France.
[4]Department of Materials, Imperial College London, London, SW7 2AZ, UK.

*Corresponding author(s). E-mail(s): x.zhou@mpi-susmat.de;

This PDF file includes:

Supplementary Table 1 to Table 3
Supplementary Figure 1 to Figure 15
Supplementary References

Supplementary Tables

Supplementary Tab. 1. Bunge Euler Angle (ϕ_1, Φ, ϕ_2) in degrees of all grains at the tilt angle of 0° in TEM.

**Grain**	ϕ_1	Φ	ϕ_2
α_1	-22.8	31.8	132.1
α_2	218.2	34.1	-32.9
α_3	222.3	55.6	143.7
α_4	135.1	25.4	-84.1
α_5	70.8	15.2	-39.3
α_6	7.5	26.5	106.6
α_7	60.2	106.7	2.7
α_8	-97.6	80.4	114.8
α_9	169.3	33.4	12.5
α_{10}	-73.0	82.2	172.6
α_{11}	-11.2	157.2	-74.1

Supplementary Tab. 2. Unit Burgers vectors of secondary GB dislocations computed for principal coincidence system of body-centered cubic (BCC) crystals

Σ values	$\mathbf{b_1}$	$\mathbf{b_2}$	$\mathbf{b_3}$
5	$\frac{a}{5}[012]$	$\frac{a}{5}[02\bar{1}]$	$\frac{a}{10}[531]$
13b	$\frac{a}{13}[13\bar{4}]$	$\frac{a}{13}[3\bar{4}1]$	$\frac{a}{26}[391]$

Supplementary Tab. 3. Misorientation axis and angle for selected CSL GBs.

GB	Misorientation Axis	Misorientation (°)	Closest CSL	θ (°) @ Axis
$\alpha_1\|\alpha_2$	[0 19 3]	$35.0 \pm 0.68°$	$\Sigma5$ [Dev: $5.3 \pm 0.68°$]	1.92 ± 0.68 @ [0 1 0]
$\alpha_3\|\alpha_4$	[20 10 -21]	$37.1 \pm 0.31°$	$\Sigma45$b [Dev: $1.1 \pm 0.32°$]	0.38 ± 0.22 @ [2 1 -2]
$\alpha_4\|\alpha_5$	[16 18 15]	$29.7 \pm 0.6°$	$\Sigma13$b [Dev: $2.3 \pm 0.6°$]	0.61 ± 0.23 @ [1 1 1]

Supplementary Figures

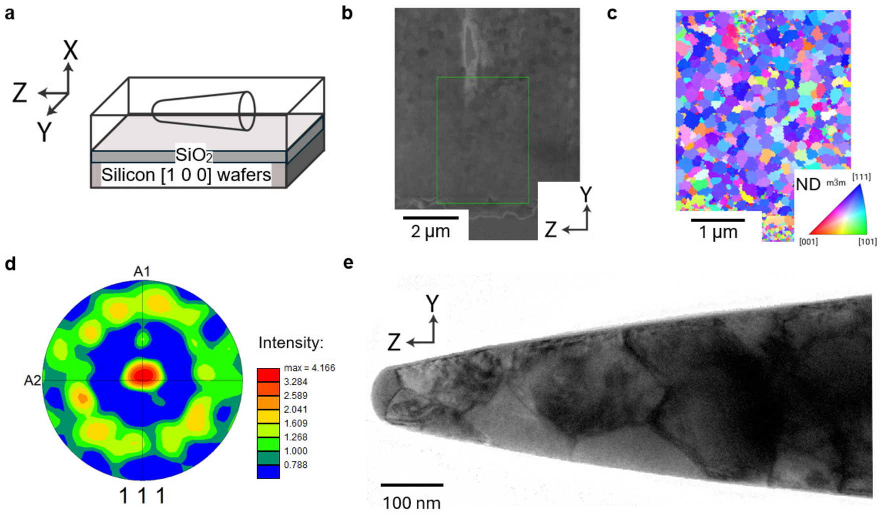

Supplementary Fig. 1. Typical microstructure of the as-prepared Fe-1 at.% W thin film. a Definition of the coordination system used to describe the geometry of the Fe-1 at.% W thin film specimen. Here, the X-axis is aligned parallel to the thin film growth direction. The Y and Z-axes, arbitrarily selected, lie in the plane perpendicular to the thin film growth direction. Once the needle-shaped specimen is prepared, the Z-axis serves as the tilting axis in TEM. **b** Region and **c** the corresponding orientation map for the transmission Kikuchi diffraction (TKD) analysis of the thin film prepared normal to the X-axis, termed as the plane view specimen. **d** (111) pole figure, oriented parallel to the thin film growth direction, for the region shown in c. **e** Bright-field image of the as-prepared Fe-1 at.% W APT sample.

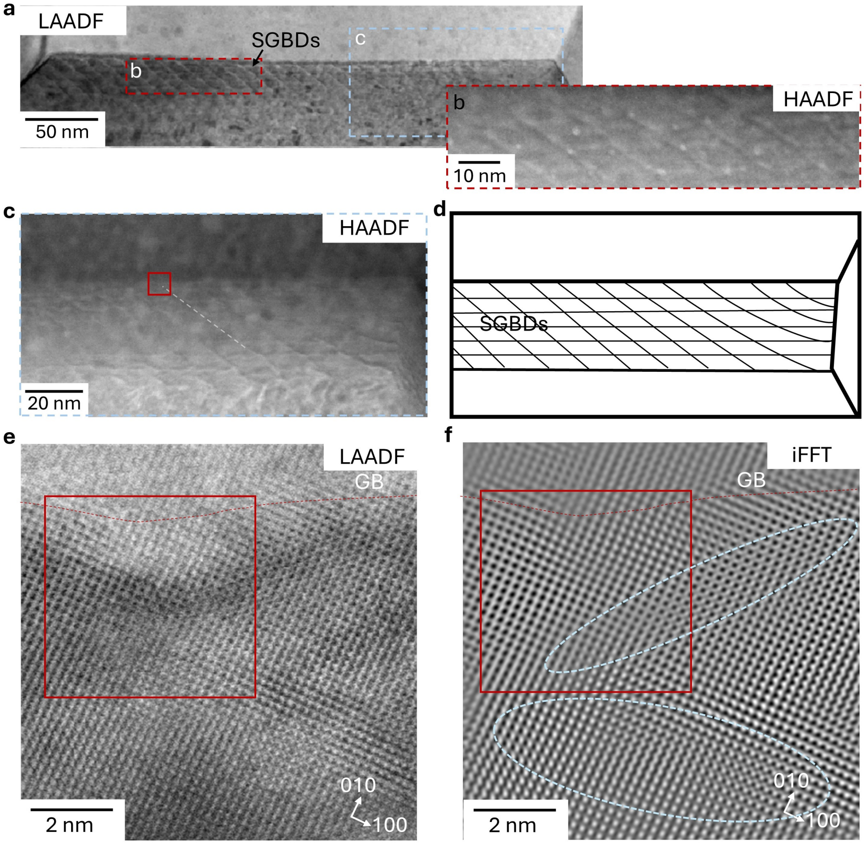

Supplementary Fig. 2. High-resolution characterization of secondary GB dislocations on the $\Sigma 11$ GB, shown in Fig. 1d. a Low-magnification low-angle annular dark-field (LAADF) image of the $\Sigma 11$ GB, highlighting periodic contrast variations associated with the secondary GB dislocation, with boxed areas indicating regions of interest for further analysis. **b & c** High-angle annular dark-field (HAADF) images of the red and blue dashed boxes shown in a, showing secondary GB dislocations along the GB plane. Variations in contrast indicate possible local compositional undulations. **d** Schematic illustration of the secondary GB dislocation network extracted from c. **e** LAADF image from the red boxed region shown in c, revealing the atomic structure near the termination of a secondary GB dislocation. The red-boxed region highlights where a secondary GB dislocation terminates. **f** Inverse fast Fourier transform (iFFT)-filtered image of the same region, showing that the presence of a secondary GB dislocation alters the local periodicity and induces lattice distortion. For this GB, Frank circuit analysis could not be performed to quantify the Burgers vector of this secondary GB dislocation. The blue dashed regions indicate the potential presence of other bulk dislocations.

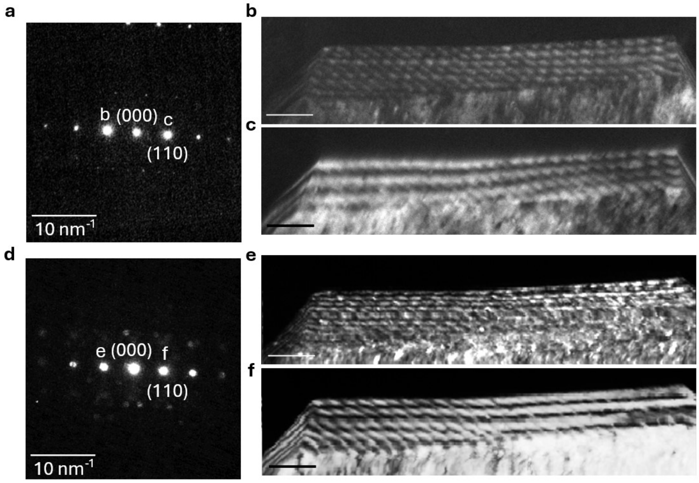

Supplementary Fig. 3. Comparison between TEM WBDF and 4DSTEM dark-field imaging for secondary GB dislocations. a Selected area electron diffraction pattern of the bottom grain in Supplementary Fig. 4 with labeled diffraction spots used for WBDF imaging. **b** & **c** WBDF images acquired under the two-beam condition using diffraction spots **b** and **c** in a, respectively, revealing the periodic contrast associated with secondary GB dislocations. **d** Converged beam electron diffraction pattern of the bottom grain in Supplementary Fig. 4, showing the spots selected for virtual dark-field image reconstruction. **e** & **f** Virtual dark-field images reconstructed using diffraction spots **e** and **f** in d, respectively. The 4DSTEM dataset obtained under the two-beam condition of spot **f** provides comparable dislocation contrast to the conventional WBDF images. Scale bar: 50 nm.

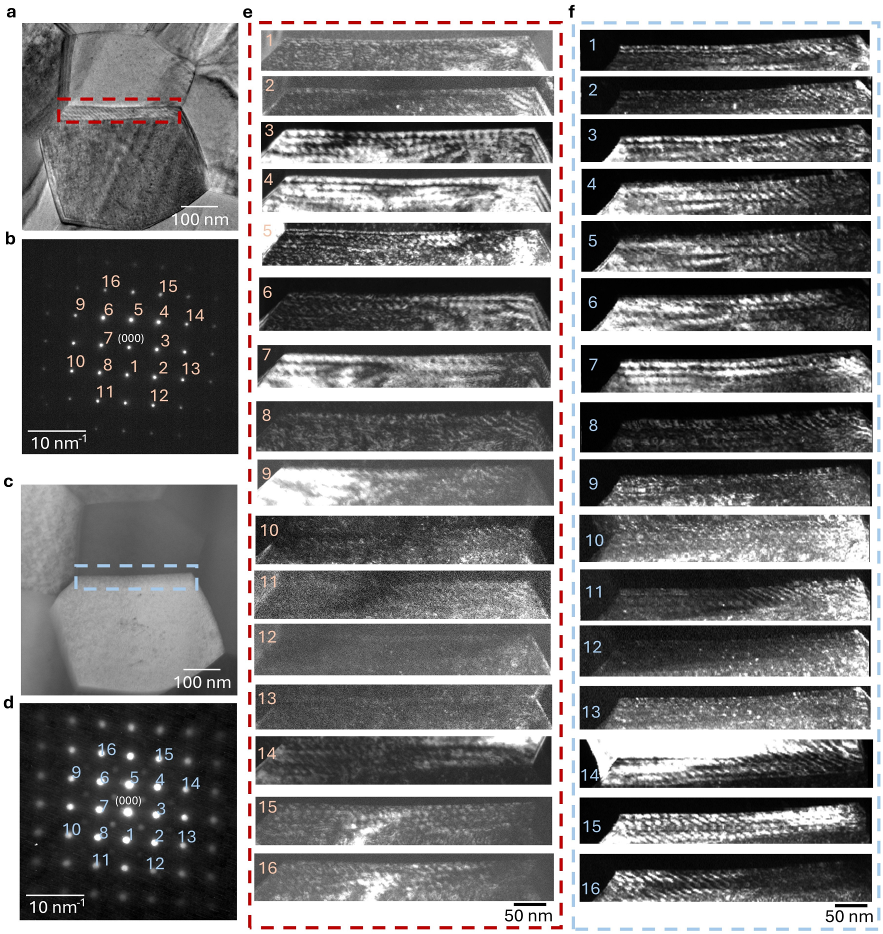

Supplementary Fig. 4. Comparison between conventional dark-field and virtual dark-field imaging of the same GB, including two associated image series. a Bright-field TEM image of the Σ11 GB shown in Fig. 1d with the dashed red box indicating the area used for the image series in e. **b** Selected area electron diffraction pattern corresponding to the bottom grain in the dashed red box in a. **c** Bright-field STEM image of the Σ11 GB region, with the dashed blue box showing the area analyzed in f. **d** Converged beam electron diffraction pattern from the bottom grain in the dashed blue box in c. **e** Conventional dark-field images corresponding to individual diffraction spots labeled 1 to 16 in b, acquired across the dashed red boxed region in a, revealing contrast variations along the GB. **f** Virtual dark-field image series based on individual diffraction spots labeled 1 to 16 in d, obtained from the dashed blue boxed region in c.

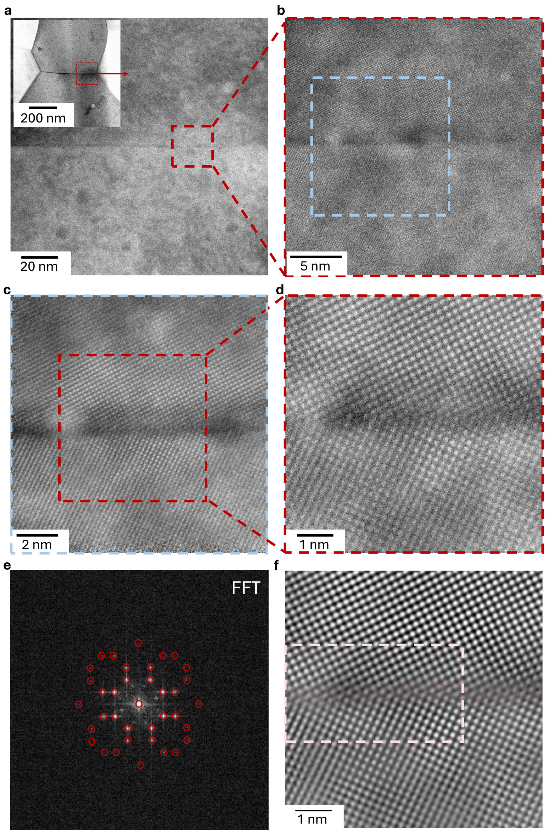

Supplementary Fig. 5. Atomic-scale imaging of a GB region containing secondary GB dislocations. a Low-magnification STEM image showing the GB region of interest. The dashed red box highlights the region selected for high-resolution imaging, as shown in b. Inset: a larger field of view indicating the GB location. **b** High-resolution HAADF image of the selected region in a. **c** Magnified view of the region indicated by the dashed blue box in b. **d** Further magnification of the dashed red boxed region in c, clearly resolving atomic columns and revealing structural distortion at the GB. **e** Fast Fourier transform (FFT) of the high-resolution STEM image in d. Red circles indicate the selected reflections used for inverse filtering. **f** iFFT image reconstructed using the selected reflections from e. The pink dashed box indicates the region used in Fig. 1k for the secondary GB dislocation analysis.

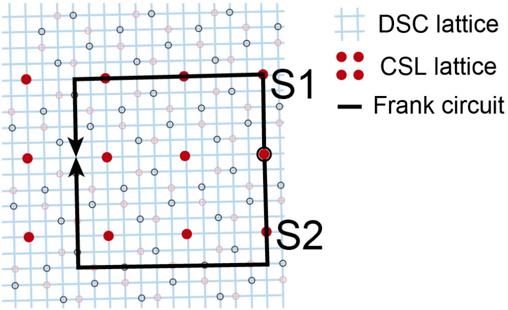

Supplementary Fig. 6. The defect-free reference Frank circuit of the $\Sigma 5$ GB corresponding to the image shown in Fig.1l. The circles represent atomic positions of the upper grain (gray) and the lower grain (pink). CSL: coincident site lattice. DSC: displacement shift complete.

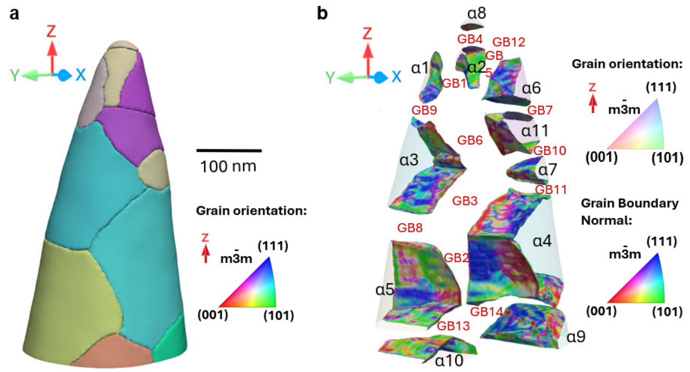

Supplementary Fig. 7. 3D crystallographic reconstruction of grains and GBs in the correlative Fe-1at.%W needle-shaped specimen. a The orientations of grains and **b** the crystallographic information of GBs, both from the same sample as shown in Fig. 2c. In b, the local normal to the GB plane has been added, and grains have been manually detached to enhance clarity. Each GB features two plane normal mappings between adjacent grains. The coordinate system for each GB plane normal mapping is referenced to the respective grain involved in the mapping.

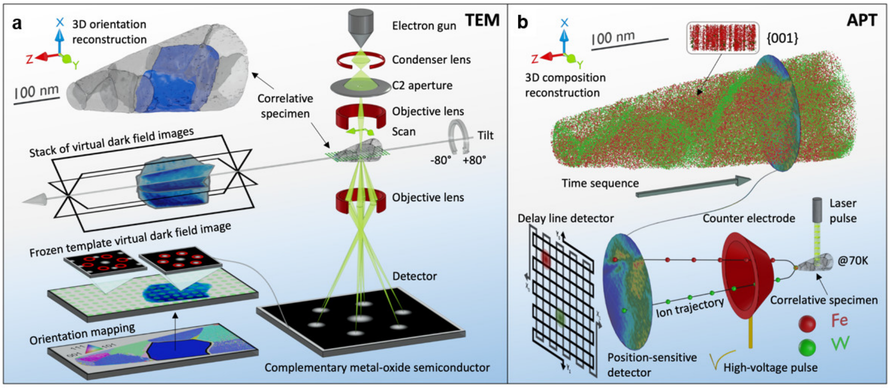

Supplementary Fig. 8. Schematics of the correlative tomography characterization techniques. **a** View of the internal optics, not to scale, inside a TEM column optimized for 4DSTEM data collection. The process involves collecting multiple 4DSTEM datasets at various tilts to generate a series of virtual dark-field images for all grains using the frozen template algorithm [1, 2]. Employing our in-house developed SPED3D software [1, 2], we achieved 3D reconstructions, with the phases and orientations of all grains quantified. **b** View of the internal setup, not to scale, inside an APT analysis chamber configured with a straight flight-path. Following TEM analysis, the specimen is transferred to the chamber and undergoes field evaporation ion by ion, with the coordinates and mass-to-charge ratio of each ion recorded for detailed 3D reconstruction and chemical analysis. X_0, X_1, Y_0 and Y_1, times obtained from the delay-line detector used to determine the ion impact positions. All illustrations in this figure were created using Blender software [5].

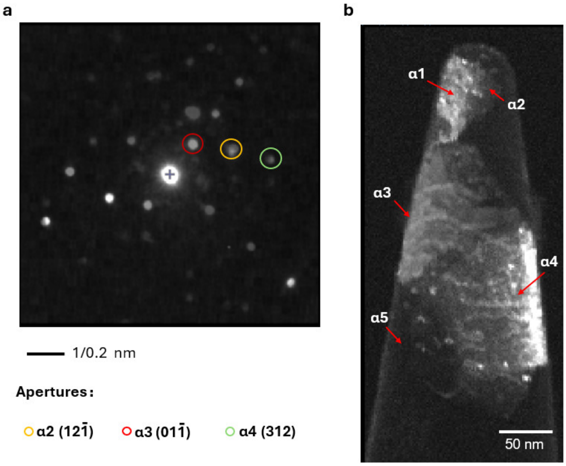

Supplementary Fig. 9. Dislocation analysis for the Fe-1 at.% W specimen. a Displays the overlaid nanobeam diffraction patterns of grains α_2 to α_4. **b** Shows a reconstructed 4DSTEM virtual dark-field image created using the virtual aperture depicted in a, which includes potential diffraction spots calculated from grains α_2 to α_4. The 4DSTEM dataset captures nanobeam diffraction patterns at each scanning pixel, allowing for the use of a virtual aperture to select one or multiple spots from the recorded patterns to reconstruct virtual dark-field images [3]. The aperture set shown in a includes the $\mathbf{g} = [\mathbf{12\bar{1}}]$ spot of grain α_2 (orange circle), the $\mathbf{g} = [\mathbf{01\bar{1}}]$ spot of grain α_3 (red circle), and the $\mathbf{g} = [\mathbf{312}]$ spot of grain α_4 (green circle), effectively highlighting grains α_2, α_3, and α_4. The diffraction pattern in a for grain α_3 illustrates a close two-beam condition. The corresponding virtual dark-field images for $(\mathbf{01\bar{1}})$ reveal the contrast of dislocations, with Burgers vectors satisfying $\mathbf{b} \cdot \mathbf{g} \neq 0$ [4]. We further analyze the line contrast on grain α_4 in b using the $\mathbf{b} \cdot \mathbf{g} \neq 0$ criterion, confirming that the line contrasts are due to dislocations.

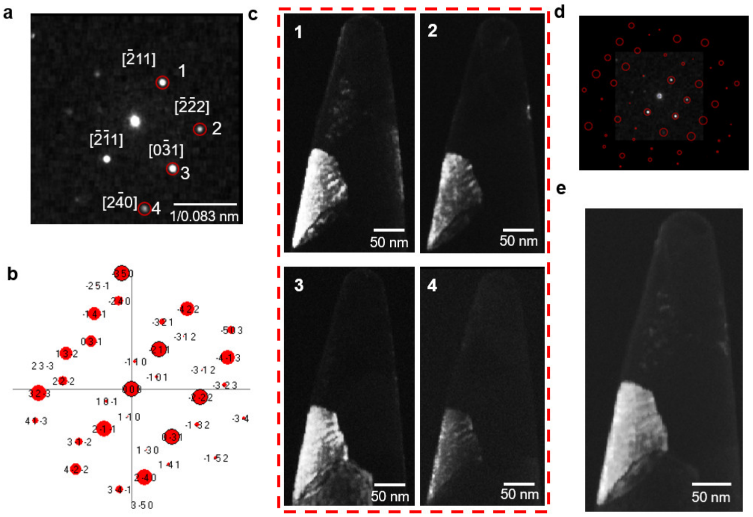

Supplementary Fig. 10. Virtual dark-field reconstruction from the 4DSTEM dataset for grain α_5. a Diffraction pattern of grain α_5. **b** Indexing of the diffraction pattern shown in a. **c** Corresponding 4DSTEM dark-field images of the virtual aperture shown in a. **d** Template-aperture set based on the crystal orientation of grain α_5. **e** Template-aperture dark-field images.

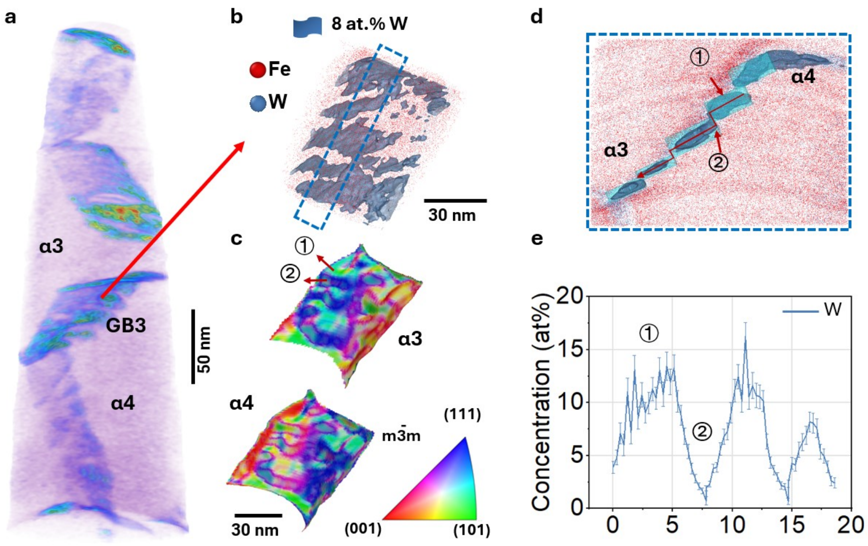

Supplementary Fig. 11. Characterization of GB facets and their link to segregation patterns in the Fe-1 at.% W specimen. a The same mapping as in Fig. 3a. **b** The isosurfaces superimposed at 8.0 at.% W on the atom maps of Fe and W highlight the GB indicated by the red arrow in a. **c** Mapping of the local normal to the GB plane between adjacent grains, from the grain α_3 side and the grain α_4 side, respectively. **d** Side view of the GB within the blue dashed-line frame shown in b, illustrating a staircase GB segregation pattern and indicating GB facets. The superimposed arrays of atoms represent the crystalline lattices for grains α_3 and α_4. **e** Compositional profile of W along the red arrow in d. The numbers ① and ② in c-e indicate the same positions across different plots.

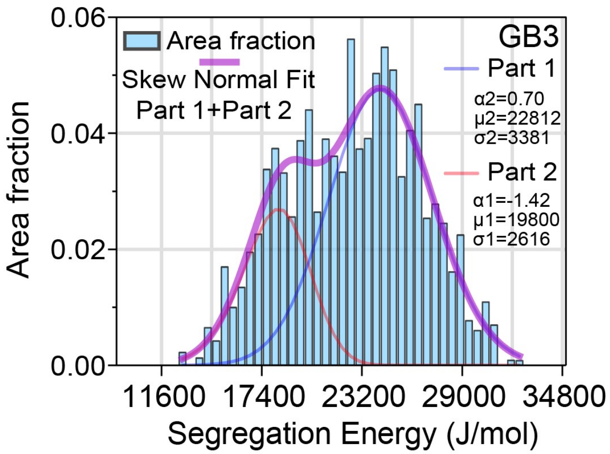

Supplementary Fig. 12. Experimental GB segregation energy spectrum for GB3. The fitting curves, overlaid on the histograms, generally follow a skew-normal distribution [6]. For GB3, the skew-normal distribution splits into two distinct domains: Part 1 for ① and Part 2 for ② observed on GB3 (Supplementary Fig. 11).

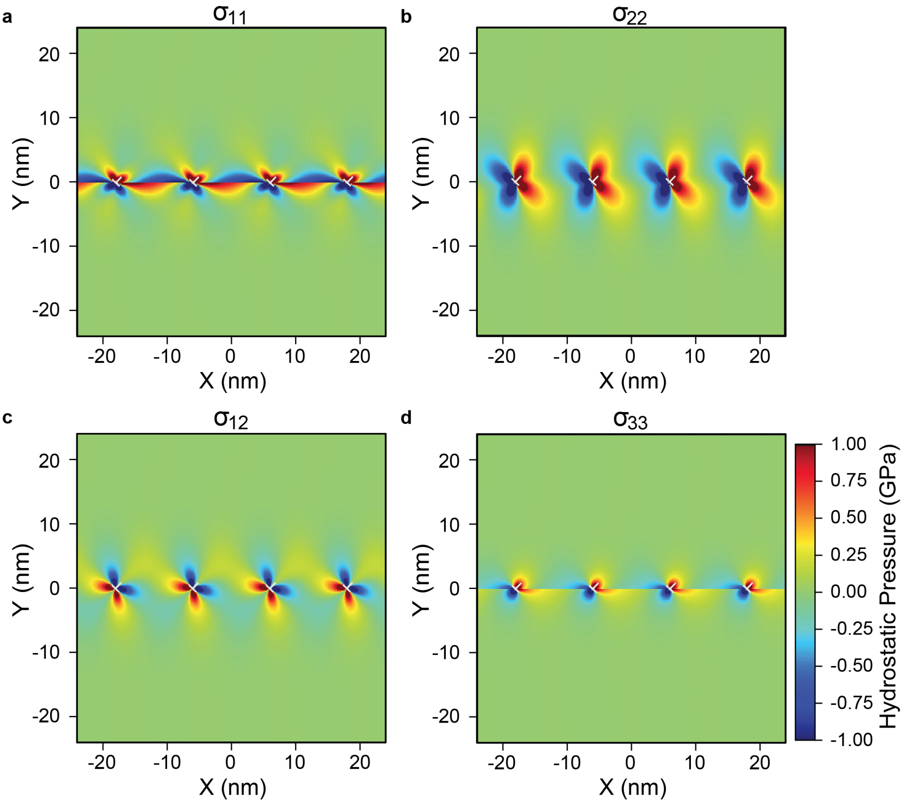

Supplementary Fig. 13. Stress component distribution analysis for the $\Sigma 13b$ GB with a DSC Burgers Vector of $\frac{a}{13}[\bar{1}4\bar{3}]$: a σ_{11}, b σ_{22}, c σ_{12}, and **d** σ_{33}.

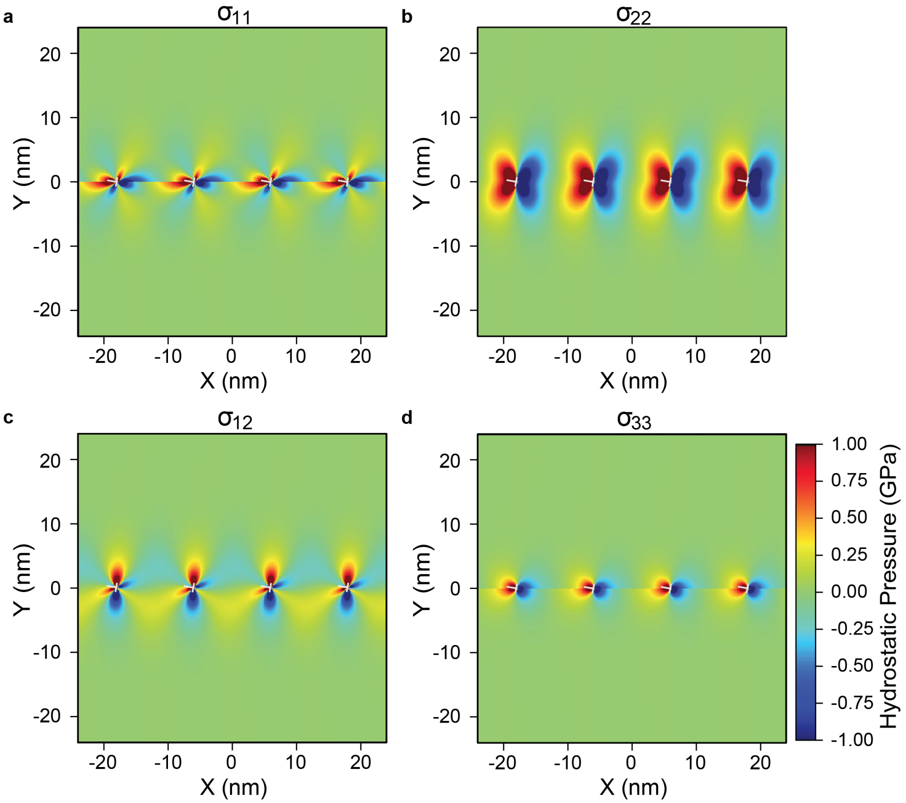

Supplementary Fig. 14. Stress component distribution analysis for the $\Sigma 13b$ GB with a DSC Burgers Vector of $\frac{a}{13}[\bar{3}\bar{1}4]$: a σ_{11}, **b** σ_{22}, **c** σ_{12}, and **d** σ_{33}.

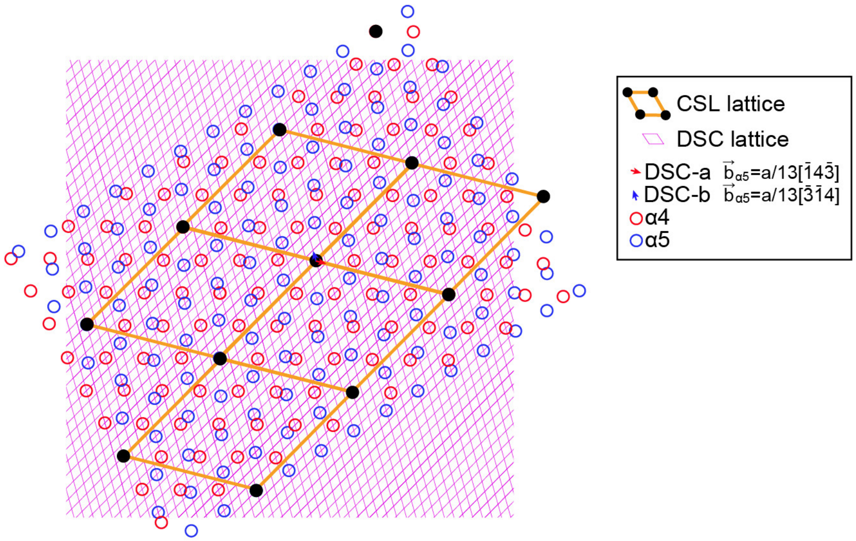

Supplementary Fig. 15. The coincident site lattice (CSL) and DSC lattice of the $\Sigma 13b$ GB. The CSL lattice is represented by black dots, forming a foundational grid. Overlaying this, the DSC lattice is depicted by pink lines. The red and blue circles signify atomic positions for $\alpha 4$ and $\alpha 5$, respectively. The red and blue arrows indicate the Burgers vectors of DSC-b, $\frac{a}{13}[\bar{3}\bar{1}4]$, and DSC-a, $\frac{a}{13}[\bar{1}4\bar{3}]$, respectively.

Supplementary References

[1] Harrison, P., Zhou, X., Das, S.M., Lhuissier, P., Liebscher, C.H., Herbig, M., Ludwig, W., Rauch, E.F.: Reconstructing dual-phase nanometer scale grains within a pearlitic steel tip in 3D through 4D-scanning precession electron diffraction tomography and automated crystal orientation mapping. *Ultramicroscopy* **238**, 113536 (2022)

[2] Harrison, P., Das, S.M., Goncalves, W., da Silva, A., Chen, X., Viganò, N., Liebscher, C.H., Ludwig, W., Zhou, X., Rauch, E.F.: Determination of five-parameter grain boundary characteristics in nanocrystalline Ni-W by scanning precession electron diffraction tomography *Ultramicroscopy* **267**, 114038 (2024)

[3] Rauch, E.F., Harrison, P., Zhou, X., Herbig, M., Ludwig, W., Véron, M.: New features in crystal orientation and phase mapping for transmission electron microscopy. *Symmetry* **13**(09), 1675 (2021)

[4] Rauch, E.F., Véron, M.: Analyzing dislocations with virtual dark field images reconstructed from electron diffraction patterns. *Microscopy and Microanalysis* **20**(S3), 1456–1457 (2014)

[5] Community, B.O.: Blender - a 3D Modelling and Rendering Package. Stichting Blender Foundation, Amsterdam (2018)

[6] Wagih, M., Larsen, P.M., Schuh, C.A.: Learning grain boundary segregation energy spectra in polycrystals. *Nature communications* **11**(1), 6376 (2020)
