# Supplementary material for: Secondary grain boundary dislocations alter segregation energy spectra
Source: Nat Commun. 2025 Sep 25;16:8422. doi: 10.1038/s41467-025-64265-6 (PMC12462497; doi:10.1038/s41467-025-64265-6)
Supplement: Supplementary file 2 — Description of Additional Supplementary Files [file 41467_2025_64265_MOESM2_ESM.pdf]

Description of Additional Supplementary Files

File Name: Supplementary Movie 1

Description: Animation illustrating the process of collecting and reconstructing data in four-dimensional scanning transmission electron microscopy tomography for material characterization.

File Name: Supplementary Movie 2

Description: Animation illustrating the threedimensional crystallographic reconstruction of the grains in the correlative Fe-1at.%W needle-shaped specimen. The color coding for the grain orientation refers to Supplementary Fig. 7.

File Name: Supplementary Movie 3

Description: Animation illustrating the threedimensional crystallographic reconstruction of the grain boundaries in the correlative Fe-1at.%W needle-shaped specimen. The color coding for the grain boundary plane normal refers to Supplementary Fig. 7.
